# A Sports Practitioner’s Perspective on the Return to Play During the Early Months of the COVID-19 Pandemic: Lessons Learned and Next Steps

**DOI:** 10.1007/s40279-021-01503-z

**Published:** 2021-09-13

**Authors:** George T. Chiampas, Abiye L. Ibiebele

**Affiliations:** 1 Feinberg School of Medicine (Emergency Medicine), Northwestern University, Chicago, IL USA; 2 McGaw Medical Center of Northwestern University (Emergency Medicine), Chicago, IL USA

## Abstract

With high profile events such as sporting and mass gathering events, recent history has revealed the importance of developing incident command structures to streamline communication, maximize coordination and establish contingencies. With the advent of COVID-19, a virus with significant human-to-human transmission and the potential for super-spreader events, there has been a brief universal cessation of sports, and the main question now is how to return to play in a way that keeps our athletes and general population healthy. This review aims to describe the core principles regarding return to play using a focus on incident command centers and disaster management. These principles include appropriate hygiene and social distancing, use of masks, rigorous monitoring and screening of symptoms, widespread testing, comprehensive contact tracing and considerations for travel and facilities. In addition, organizations need to have established scalable protocols for athletes who do contract the virus with symptom-based algorithms for length of time away from play and with screening for cardiac and pulmonary complications from COVID-19. Also, encouraging our athletes to become immunized against the virus and educating our athletes about nutrition and the relation to immune health is important as we return to play.

## Key Points

**Table:** 

COVID-19 provides new challenges and considerations as we consider how to return to play.
A successful return to play requires an emphasis on physical distancing, masks, symptom monitoring, testing, contact tracing and considerations for travel and facilities.
Using an incident command structure, organizations can streamline communication and be successful in incorporating the above principles.

## Introduction

With the onset of the Coronavirus Disease 2019 (COVID-19) pandemic, a new virus was introduced to the world community with significant human-to-human transmission and wide-ranging impacts on population health and the ability of groups of people to gather together. However, it is important to note that throughout the history of sports, there have been instances where external events and factors posed a danger to the safety and security of the participants, onlookers, and the public. Some of these events have been man-made, such as in recent history the bombing of the Boston Marathon in 2013 or the 2015 Paris Terror Attacks which impacted the France vs Germany soccer match. More commonly, the causes of safety threats are due to more natural causes, such as inclement weather causing a game or event to be postponed or a high heat index leading to potentially dangerous conditions for athletes to be training or competing in. While these events affect sports of all levels, the popularity and scale of larger events require planners to prepare for possible complications, last minute mitigation decisions, and changes to procedures that may arise with these natural threats. As we reflect on our initial actions in the COVID-19 pandemic and continue to move forward in our response to COVID-19, the importance of effective communication and a clear command structure have become apparent. Here we provide a narrative review of return to play during the COVID-19 pandemic, starting with an overview of incident command, followed by a description of COVID-19 illness, its impact on the sports world, and finishing with a discussion on return to play while focusing on keeping athletes and populations safe and healthy.

### Incident Command

To manage high profile events, it is important to recognize that these events could become disasters and to set up a command structure to manage them as such. In the United States, after 2001, the United States Department of Homeland Security developed the National Incident Management System [1].

The goal of the system is to provide a “consistent nationwide approach for federal, state, tribal and local governments to work together to prepare for, prevent and respond to and recover from domestic incidents, regardless of cause, size or complexity” [1]. This led to the more widespread use of an “Incident Command Structure (ICS)”. An ICS is defined as a management system designed to enable effective and efficient incident management by combining facilities, equipment, personnel, procedures and communication in one organized structure [2].

One example of how ICS has become integral to the success of sporting events is the Chicago Marathon. In 2007, the marathon was canceled due to high race day temperatures; however the decision to “cancel” was not clearly predefined. This led to confusion in terms of procedures and logistics so that an individual police officer did not know what instructions to give race participants as to whether they could walk the rest of the race or if they had to be cleared from the city streets [3]. However, a greater adoption of the ICS led to increased coordination in the following two years. The marathon was viewed by the planners and medical committee as a “Planned Mass Casualty Event,” in the sense that it had the potential to be similar to a disaster in terms of the scope of potential people affected and strain on local resources and infrastructure. The key difference is that planning and medical committees have months to prepare for possible challenges and complications and have the ability to set disaster plans, command structures and contingencies in advance.

## COVID-19

With its impacts on the sporting and entertainment world and public gatherings, COVID-19 has led to unique challenges when it comes to sporting events and mass gatherings. When it was first described in the literature after the initial outbreak in Wuhan, China, COVID-19 was described as a virus with significant human-to-human transmission with each infected person spreading the virus to at least two other individuals and the possibility for super-spreader events [4]. It was thought to be spread by respiratory droplets as well as direct contact, with an observed death rate at the time between 1.4–3.2% [5]. We now know that COVID-19 primary method of spread is through respiratory droplets; however in certain situations, such as enclosed spaces without proper ventilation, the virus can be spread through an airborne route [6]. The most current data by the World Health Organization (WHO) suggests that 80% of people who contract COVID-19 recover without the need for hospital treatment, 15% become seriously ill and require oxygen and 5% become critically ill and require intensive care [7]. Patients who are 60 years or older or who have significant medical comorbidities are more likely to become seriously ill. However, there also have been deaths from COVID-19 seen in younger individuals with no other medical comorbidities. Numbers so far have shown it to be a more severe respiratory virus when compared to influenza, which averages between 290,000 and 650,000 deaths per year worldwide out of an estimated 1 billion cases leading to a death rate between 0.029% and 0.065% [8].

There are treatments available for COVID-19 once someone becomes infected; however these treatments have minimal effect on health outcomes of patients who do not require hospitalization and do not have a significant effect on spread of the disease [9, 10].

### Impact on Sports

With regards to sporting events and public gatherings, as the disease progressed in early 2020 to officially being declared as a pandemic on March 11, 2020, it became clear that team physicians and the medical committees of organizations would need to address COVID-19 as it related to their sports. In the authors’ experience, it became important to coordinate this response in the framework of an incident command center as we were all learning about the virus together with the ideal that we would also make decisions together, across multiple stakeholders, organizations, leagues and sports. With this, came the importance of unified messaging with single answers and to “get things right” the first time, to avoid confusion of volunteers, athletes, coaches and event planners. However, it also became important to come to a decision and proceed in a unified manner once a decision had been made.

Around the time that the WHO was making the decision to declare the current outbreak of COVID-19 as a pandemic, multiple organizations all throughout the world of sports were ending their seasons early or deciding to postpone or cancel plans for seasons which were about to begin, even including the Olympic Games scheduled for the summer of 2020. Once this happened, the goal and the question shifted to how we begin to re-enter and restart sports again in a way that keeps our athletes, coaches, staff and public safe.

## Return to Play

When considering return to play, it is important to focus on protocols that take into account the systems in place as well as individuals. From a system and organizational standpoint, return to play is the process of restarting events, with COVID-19 measures in place. For this to happen, it is important for sports organizations to expand the concept of team. A team not only includes the athletes or competitors of a soccer team, for example, but must also include the coaches, managers, athletic staff, medical staff, support staff as well as other members of the organization. While the team concept seems basic, the COVID-19 pandemic shed a greater light on the interconnectivity of all members of the delegation. A member of any of these groups may contract the virus and spread it to other members or teams, which could lead to an outbreak, limiting the ability of the team and league to play and succeed.

### Pillars for Return to Play

To minimize the risk of COVID-19 transmission, any organization’s plan for return to play should include the following six pillars.

1. Appropriate hygiene and physical distancing
2. Use of masks
3. Rigorous monitoring and screening of symptoms
4. Widespread testing
5. Comprehensive contact tracing
6. Considerations for travel and facilities

#### Appropriate Hygiene and Physical Distancing

An essential method of preventing the spread of COVID-19 is to ensure our athletes are washing their hands appropriately and as frequently as possible [11]. Athletes should be instructed on how to wash their hands appropriately and to avoid contact with their faces and mouth after having contact with a surface that could possibly be contaminated. While soap and water are superior to hand sanitizers, an alcohol-based hand sanitizer containing at least 60% alcohol is an appropriate alternative [12]. In addition, physical distancing can help reduce contacts between any possible athletes who have been infected with the virus but are not symptomatic and those who have not been exposed to the virus [11]. Physical distancing includes keeping 6 feet in between athletes when possible, limiting non-essential visitors and spectators, and avoiding congregating in large groups when possible [13].

#### Use of Masks

In addition to an emphasis on hygiene and physical distancing, another item in our toolbox to prevent the spread of COVID-19 is the use of masks. It is known that the use of masks helps reduce the spread of COVID-19 between individuals [14]. All individuals should wear a mask that covers their nose and mouth [13]. A cloth mask is appropriate to avoid spread in the sports setting [14]. With higher intensity sports and competition, it may not be feasible for athletes to wear masks. In these instances, the organization should work to make sure sports are taking place either outside or in a well-ventilated area [13].

#### Rigorous Monitoring and Screening of Symptoms

Given the possibility of COVID-19 to rapidly spread amongst a team, it is important to rapidly identify individuals who may be infected with the virus. Given this, athletes should be screened daily for possible symptoms of COVID-19. The limitation in this method is that it will not detect athletes who have asymptomatic or subclinical presentations of COVID-19. However, it can be used in conjunction with the other pillars described. Self-reported symptom tracking through mobile applications has proven an effective and efficient way to screen for symptoms daily [15].

#### Widespread Testing

In addition to rigorous monitoring and symptom screening, widespread testing is important to detect the athletes who have asymptomatic or subclinical presentations of COVID-19. While the ability of different organizations to test depends on the level of sport and resources, at minimum, all athletes should be tested prior to the start of the season. Simulations for airline travel show that polymerase chain reaction (PCR) testing 72 h prior to travel is an effective testing strategy for asymptomatic travelers [16]. This 72-h timeframe has been widely adopted by different cities, states and countries with regards to allowing visitors in. We recommend a minimum similar strategy for testing in non-vaccinated individuals prior to the beginning of a sporting match or event. Ideally, serial testing would also take place prior to competition between teams but this should be guided by accessibility and costs as well as organizational resources. Management of athletes who test positive is further discussed in the section below regarding protocols for athletes after infection.

#### Comprehensive Contact Tracing

If and when athletes do test positive, it is important for organizations to have protocols in place for contact tracing, which involves identifying individuals who may have been potentially exposed to the virus and informing them of their need to isolate. Organizations should be meticulous in maintaining a list of everyone who is present at team events or in the facility for contact tracing purposes. Depending on the level of sport or the event, there is also an opportunity for the use of technology to aid with contact tracing. The use of Bluetooth technology, mobile applications or global positioning systems (GPS) bracelets may be able to be used to identify athletes and staff who are “high risk” contacts with a person who tests positive [17].

#### Consideration for Travel and Facilities

COVID-19 has created new challenges with regards to the physical spaces where we take part in sports as well as how we travel. It is important to clean and disinfect highly trafficked areas of the facility and it may be helpful to enact one way traffic areas in the facility to ensure physical distancing. Wherever possible, maintaining 6 feet of distance between individuals can help prevent spread of infection and flexibility in how we use our spaces can help achieve this. With regards to travel, physical distancing is important as well. Travel should only take place if the overall rates of infection for both the team and community are low and should happen with respect to directives from health officials. For team travel, accommodations should be made to ensure appropriate distancing between athletes which may include increasing the number of vehicles or reducing the number of individuals travelling.

### Models of Return to Play

An example of the pillars being implemented successfully was the Bundesliga professional football (soccer) league in Germany [18]. After the onset of the pandemic, they appointed a task force to develop a hygiene protocol based on strict hygiene, physical distancing rules, symptom screening and repeated testing. They subsequently restarted their two highest men’s leagues and reported on their experience in the literature [18]. In these leagues, all players were required to complete a daily symptom screening questionnaire in a mobile application. In addition to players, other groups of individuals including ball boys, referees, doping control officers and TV personnel also had to complete daily questionnaires. All teams also had a designated hygiene officer who was a medical doctor. All players were required to have two negative swabs for SARS-CoV-2 within 5 days from the beginning of training camp, and afterwards had two viral PCR tests performed every week. Of note, referees were also included in PCR testing. If there was a match in a given week, one test would be performed within 24 h of the match. Ultimately 1702 individuals were tested. Eight players and four officials tested positive prior to training camp, and they were put into quarantine. After training camp began, two more players tested positive during the third round of testing, with no further positive test results for the rest of the season which started on May 16, 2020 and ended July 6, 2020.

Another model that has been utilized is the bubble concept, most famously employed by the National Basketball Association (NBA). This included an initial suspension of the season followed by a re-introduction into an isolated environment. Similar to the German model, it is reported that players had to test negative twice to enter the bubble [19]. They then had strict rules limiting player socialization with other teams and had to quarantine if any player left the bubble. Of note, there were zero positive cases of COVID-19 for the duration of the NBA season in the bubble [20]. There are also other models that have been utilized which include having a less restrictive “controlled environment” with variations in testing frequency, with decreased success in preventing spread of COVID-19 in athletes [21]. The controlled environment is one that is more sustainable in sport where players and staff live in their home environments and continue to participate with screening, behavioral modifications and serial testing. Of note, the first professional sport in the United States to proceed in 2020 was the National Women’s Soccer League (NWSL). The NSWL hosted the “Challenge Cup” in June 2020 in a bubble format similar to the NBA, which highlights the collaborations across leagues and medical officers operating as “Incident Commanders” during the pandemic.

In addition to protocol and operational changes, it is imperative for every organization to be in tune with directives by state and local health officials. If the prevalence of COVID-19 in the community is higher than what regional health officials deem is safe, then in the absence of a completely isolated environment, members of the team may contract the disease outside of sporting activities and cause outbreaks amongst the team. Thus, it is important for organizations to adjust their operations in response to directives from health officials.

### Youth Sports

The level of sport that has the highest risk of athletes contracting the disease outside of sporting activities is youth sports. The majority of youth sports are tied to schools, and a significant number of children are involved in sports through school. With school closures and the transition to virtual learning, access to in-person activities is dependent on local health directives and school boards. Since the onset of COVID-19, what has been observed is a transition away from organized sports and sports practices [22].

One challenge with youth sports is that there is not sufficient funding or resources to perform widespread testing at the levels seen with professional sports. A youth club in Washington state, Seattle United, reported a model in which they divided their team into pods of five individuals for practice with an emphasis on symptom monitoring, wearing masks at all times when not exercising and physical distancing, including during training [23]. Their efforts led to only 2 players out of the 15,494 who attended practices over the course of about 1 month testing positive. This is notable because the club did not have the capability to test players but instead encouraged athletes to get tested if they developed symptoms. Most other youth sports clubs or school sports will similarly not have the capability to offer their athletes tests, so this study is important in that it shows a model for keeping athletes safe and healthy with a focus on distancing, hygiene, masking and symptom monitoring. This strategy, however, is dependent upon the ability of athletes to get tested if they do develop symptoms which requires widespread access to testing in the community. As such, in order for youth sports to reemerge at pre-pandemic levels, communities must increase their testing capabilities to ensure widespread and quick testing. U.S. Soccer developed their return to play pathway for COVID-19 (http://www.ussoccer.com/playon) across all levels of the game including youth. As the national governing body of the sport, the protocols were aimed to tie across the game domestically and to assure a singular message to international partners from players of all levels, coaches and all stakeholders of the game. Limitations of the implementation of the “COVID PlayOn” protocols were a lack of adoption and alignment across state health departments and implementation from the National Federation of High School Sports (NFHS). On future health and safety matters, these collaborations are critical for compliance and consistency within sport.

### Protocols for Athletes After Infection

The other aspect of return to play that requires attention is the development of protocols concerning athletes who have contracted the virus. Expert consensus recommendations have been disseminated by the American College of Cardiology Sports and Exercise Leadership Council based on the complication of myocarditis which can arise after COVID-19 [24]. These recommendations suggest that asymptomatic patients who test positive, after a 2-week period of isolation and no exercise, should have a slow resumption to activity under the guidance of the health care team. For athletes with symptoms who do not require hospitalization, the group recommends a 2-week period after resolution of symptoms followed by an evaluation by a health care professional which includes biomarker testing and cardiac imaging. For athletes who require hospitalization, it is recommended to have biomarker testing and imaging done while in the hospital. If these tests are unremarkable, it is recommended for the athletes to have a repeat evaluation by a healthcare professional 2 weeks after cessation of symptoms. If at any point an athlete has an abnormal cardiac test, then they should follow myocarditis return to play guidelines [25].

Similar guidelines have been published by researchers based in the United Kingdom [26]. These recommendations suggest that patients with mild symptoms who managed their symptoms at home and recovered, defined as being symptom free at rest and no sooner than day 10 from onset of symptoms, should have a thorough history and physical as well as an electrocardiogram (EKG) and echocardiogram done before return to play. If these tests are abnormal, cardiac magnetic resonance imaging (MRI) should be performed. However, these patients do not require respiratory testing. For patients with prolonged illness lasting over 14 days, cardiac MRI should be done in addition to a thorough clinical evaluation and EKG. If MRI is normal, they should have exercise testing and 24-h Holter EKG. If they have respiratory symptoms, they should also have a chest X-ray done and biomarker testing to evaluate for inflammation, myocyte necrosis and thromboembolic disease. If results are abnormal, they should have computed tomography (CT) imaging of the chest done with cardiopulmonary exercise testing if the CT is inconclusive. If athletes have had COVID-19 severe enough to be hospitalized, they should have a full cardiac and respiratory workup.

It is important to note that as the sports medicine community continues to learn more about COVID-19, recommendations and standards of practice should continue to evolve. A letter to the editor of the American College of Cardiology Sports and Exercise Leadership suggests including repeated COVID-19 testing following recovery and a symptom-based algorithm for testing with the focus again on evaluating for cardiac, as well as, pulmonary complications of COVID-19 [27]. While these are recommendations, not guidelines, this framework provides a basis for designing return to play protocols for individual athletes based on what is currently known about COVID-19 and it is important to tailor evaluation to resource availability and local standards of care. One exception is the suggestion for repeated testing of athletes after their symptoms have resolved. As our knowledge of COVID-19 has increased, it has become apparent that people who contract the virus can continue to shed detectable SARS-CoV-2 RNA but not be contagious long after they have recovered from COVID-19 [28]. These details should be considered in safe and appropriate clearance of athletes and staff to return to sports and or events.

### Vaccinations

Per the World Health Organization, as of June 2021, at least 13 different vaccines targeted against COVID-19 have been administered for a total of 1.63 billion doses [29]. Overall, vaccines have been shown to be efficacious against COVID-19 and safe, with rare side effects similar to other vaccines [30–32]. While administration thus far has been limited to adult populations, it is anticipated that soon vaccines will start to be approved for younger individuals. As vaccine supply continues to rise across the globe and becomes more evenly distributed, it is fair to anticipate a “passport to participation” comprised of a combination of being fully vaccinated and/or negative PCR testing for return to sports with a progressive release of restrictions over time as vaccinated individual percentages increase. However, while we return to play, it is important to note that vaccination does not replace the need for the six pillars of return to play described above. No vaccine is 100% efficacious against COVID-19 and with the rise of variants of COVID-19, there is a risk of illness after successful vaccination [33]. Despite this, vaccines remain our best method of protecting our athletes against COVID-19 infection and from a public health perspective, widespread vaccination is our path to achieving herd immunity to COVID-19 and our road towards the end of the pandemic.

### The Role of Nutrition

Another way we can protect our athletes from COVID-19 is by making sure we emphasize the effects that nutrition can have on the immune system, especially as it relates to this virus. Severe cases of COVID-19 (e.g., those involving respiratory distress, septic shock) are thought to be caused by a severe inflammatory response [34]. The “Western” diet, high in processed foods, deep-fried foods and high-fat products has been associated with hyperglycemia, development of an inflammatory state and metabolic complications [35]. This diet could compound negative health outcomes, especially given what we know regarding COVID-19 and inflammation. One paradigm that has been researched prior to the onset of COVID-19 is the concept of immune resistance and immune tolerance [36]. Immune resistance is the ability of the immune system to destroy microbes, while immune tolerance is the ability to control infection at a non-damaging level, to avoid harm to bodily tissues [36]. We should be encouraging and empowering our athletes and patients to consume foods that can create homeostasis between these two systems. This includes obtaining foods with an adequate supply of energy from glucose, amino acids and fatty acids to supplement the resistance of the immune system, as well as micronutrients such as vitamin C which reduces oxidative stress and vitamin D which can help mitigate against tissue damage during infection and improve immune tolerance [36, 37]. With other respiratory illnesses, zinc has been shown to decrease upper respiratory illness (URI) duration, and vitamin C has been shown to reduce illness incidence in athletes [36, 38].

Vitamin D has also been shown to enhance cellular immunity and reduce expression of pro-inflammatory signals which are associated with COVID-19 [39]. Due to studies suggesting that in athletes with URI symptoms, athletes with positive microbiology results had lower levels of vitamin D and that vitamin D supplementation also reduces URI incidence, there has been a substantial amount of research on vitamin D in the treatment of COVID-19 [40, 41]. When looking at population data of European countries, a significant correlation was observed between the mean concentration of vitamin D levels and the number of COVID-19 cases, with more cases being observed in countries with lower concentrations; however this same correlation was not seen with the number of COVID-19 deaths [42]. Other studies have also seen an association between vitamin D levels and ICU vs floor status in patients hospitalized with COVID-19 and it has been suggested that people at risk for vitamin D deficiency should take supplementation to maintain circulating 25-OH-D at an optimal level between 75 and 125 nmol/L [42, 43]. While there are still many unanswered questions including if vitamin D supplementation is better used as prophylaxis or treatment, what the optimal levels of vitamin D are, and what populations can most benefit from supplementation, it is important as sports medicine physicians that we recognize the effect nutrition can have on our athletes in the setting of the COVID-19 pandemic. We need to emphasize the need to not only condition our athletes to avoid physical injury, but also focus on conditioning and optimizing the immune system through diet to help avoid illness.

## Conclusion

In conclusion, the advent of COVID-19 has created significant challenges when it comes to sports and mass gatherings. With the potential for super-spreader events, its mode of transmission and possibility for adverse health outcomes, it has become imperative to first and foremost keep our athletes safe. In addition, we need to stress the importance of keeping the other members of the team safe, as the health of one person has the potential to affect the health of everyone. A successful return to play during the COVID-19 pandemic necessitates organizations adopting an incident command system with a coordinated approach and an emphasis on communications. It requires first a commitment to an Incident Lead such as the Team Physician and protocols focused on hygiene, social distancing, masks, monitoring/screening, widespread testing, contact tracing and with a consideration for travel and facilities. We need to make sure the sports community leads in building awareness amongst the public regarding vaccines and encourage our athletes to become immunized against COVID-19 while making sure they are educated about appropriate nutrition to optimize their immune systems and have detailed protocols for return to play for athletes who contract the virus. Most importantly, we need to address these challenges as a team. To be successful, it will require transparency, honesty, personal responsibility, compliance and sacrifice… the building blocks of all great teams.
